# Mental Experiences in Wild Animals: Scientifically Validating Measurable Welfare Indicators in Free-Roaming Horses

**DOI:** 10.3390/ani13091507

**Published:** 2023-04-28

**Authors:** Andrea M. Harvey, Ngaio J. Beausoleil, Daniel Ramp, David J. Mellor

**Affiliations:** 1 Centre for Compassionate Conservation, TD School, University of Technology Sydney, Ultimo, NSW 2007, Australia; daniel.ramp@uts.edu.au; 2 Animal Welfare Science and Bioethics Centre, School of Veterinary Science, Massey University, Palmerston North 4442, New Zealand; n.j.beausoleil@massey.ac.nz (N.J.B.); d.j.mellor@massey.ac.nz (D.J.M.)

**Keywords:** horse, welfare assessment, wild horse, free-roaming horse, wild animal, wildlife, Five Domains Model, mental experience, affective state, scientific support, welfare indicators, validation

## Abstract

**Simple Summary:**

The mental experiences of animals are what characterises their welfare status. The Five Domains Model is a contemporary holistic framework for assessing animal welfare, based on the understanding that physical and mental states are linked. Following measurement of indicators within each of the four physical/functional Domains (1. Nutrition; 2. Physical environment; 3. Health; and 4. Behavioural interactions), the anticipated negative or positive mental experiences are assigned to Domain 5. This review refers to indicators of physical/functional states in Domains 1 to 4 in free-roaming wild horses and evaluates the scientific evidence linking them to inferred mental experiences in Domain 5. We demonstrate that indicators can be assessed for a range of negative mental experiences that includes thirst, hunger, heat and cold discomfort, localised pain, non-specific chronic pain/malaise/fatigue/exhaustion, weakness, breathlessness, social pain, anxiety and fear; and also for a range of positive mental experiences, including pleasures associated with drinking, mastication, post-prandial satiety, cooling and warming, vitality of fitness, exercising agency, and participating in affiliative social interactions. This body of evidence supports the application of indicators of physical state and behaviour, measurable from free-roaming horses, to infer various mental states relevant to their welfare.

**Abstract:**

The mental experiences of animals are what characterises their welfare status. The Five Domains Model for assessing welfare aligns with the understanding that physical and mental states are linked. Following measurement of indicators within each of the four physical/functional Domains (1. Nutrition; 2. Physical environment; 3. Health; and 4. Behavioural interactions), the anticipated negative or positive affective consequences (mental experiences) are cautiously inferred and assigned to Domain 5. Those inferences derive credibility from validated knowledge of the underlying systems of physiology, neurophysiology, neuroethology and affective neuroscience. Any indicators used for assessing welfare need to be scientifically validated. This requires, firstly, evidence of the links between a measurable/observable indicator and the physical/functional impact (in Domains 1 to 4), and secondly, a demonstrable relationship between the physical/functional impact and the mental experience it is inferred the indicators reflect (in Domain five). This review refers to indicators of physical/functional states in Domains 1 to 4, which have been shown to be measurable in free-roaming wild horses, and then evaluates the scientific evidence linking them to inferred mental experiences in Domain 5. This is the first time that the scientific evidence validating a comprehensive range of welfare indicators has been synthesised in this way. Inserting these indicators into the Five Domains Model enables transparently justifiable assessment and grading of welfare status in free-roaming horses.

## 1. Introduction

**Figure 1:**
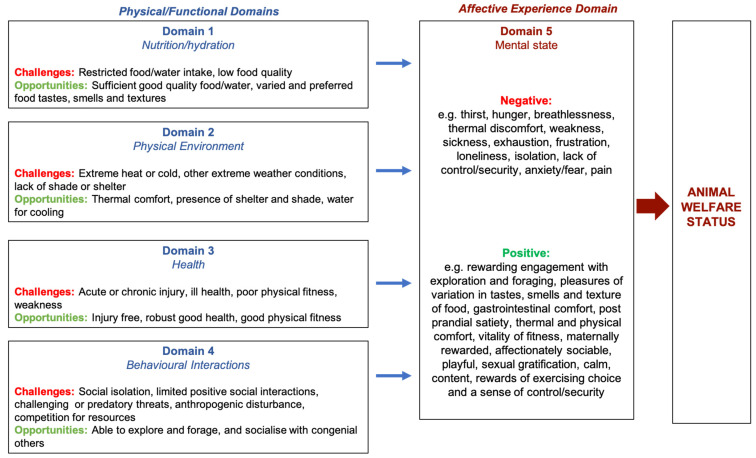
An abbreviated schema of the Five Domains Model (adapted from Harvey et al., 2020 [2]), showing negative and positive physical/functional states or situations (Domains 1 to 4) and examples of their associated negative and positive mental experiences or affects (Domain 5), relevant to free-roaming wild horses. Taken together, these mental experiences represent the overall welfare state of the animal.

Contemporary animal welfare science aims to interpret indicators of biological function and behaviour in terms of the mental experiences that those indicators are likely to reflect [1,2,3]. The Five Domains Model for assessing welfare status has been described in detail [2,3,4,5,6,7] and is illustrated again here (Figure 1). It structurally represents the understanding that physical and mental states are linked and facilitates the assessment of welfare based on our current understanding of the functional bases of negative and positive subjective experiences that animals may have. Following measurement of indicators within each physical/functional Domain, anticipated negative or positive affective consequences are cautiously inferred and assigned to Domain 5. It is these mental experiences that contribute to descriptions of the animal’s welfare state [1,2,3].

These mental experiences are subjective and therefore cannot be measured directly. However, their presence or absence, and sometimes severity and duration, can be inferred. Such inferences derive credibility from validated knowledge of the underlying systems of physiology, neurophysiology, neuroethology, and affective neuroscience, and also from the caution exercised when inferring the presence of specific affects [2,3,4,7]. Three points must be kept clearly in mind when using the Model to infer subjective experience: (1) it is the indicators of the animal’s physical functional states that are measurable, not the animal’s mental experiences; (2) it is the credible links between these indicators and identifiable mental experiences of welfare significance that determine which indicators have the most utility in specific contexts; and (3) it is these selected indicators that should be prioritised to practically assess animal welfare [2].

A Ten-Stage Protocol for applying the Five Domains Model for assessing welfare, using free-roaming wild horses as an example, has been published (summarised in Table 1) [2]. Stage 3 of the Protocol, which requires reviewing the current knowledge of free-roaming wild horses within each of the four physical/functional domains, has also been published [8]. This knowledge underpinned Stages 4 to 6 of the Protocol in which novel methods for practically measuring/observing a range of animal-based welfare indicators in free-roaming horses were developed, evaluated, and described [9].

Stage 7 of the Protocol involves scientific validation of those putative indicators of welfare. This requires scientific evidence of the links between each measurable/observable indicator and it’s measurable physical/functional impact (in Domains1 to 4), as well as scientific validation of the relationship between each physical/functional impact and the mental experience(s) it is proposed to reflect (in Domain 5).

Stage 8 involves applying the Model welfare assessments and grading using only those indicators that are scientifically supported, with the additional precaution at Stage 9 of assigning confidence scores that reflect the degree of certainty about the data upon which the grade was based. Finally, at Stage 10, in addition to these animal-based ‘welfare status indicators’, other indicators can be considered. These ‘welfare-alerting indicators’ can be animal or resource based but do not directly reflect an animal’s mental state; rather, they indicate the *risk* that welfare may be impacted negatively or positively, either at a previous time, the current time, or in the future. For example, we are alerted to the risk of animals’ previous thermal discomfort by historical records of cold weather or to the risk of future thermal discomfort if cold weather conditions persist [2].

The aim of this paper is to scientifically validate previously described practical and measurable/observable welfare indicators for free-roaming horses and to distinguish between ‘welfare status’ and ‘welfare alerting’ indicators [9]. Validated indicators can then be inserted into the Five Domains Model to enable the assessment and grading of welfare status in free-roaming horses (i.e., their subjective mental experiences). This is the first time that the scientific evidence validating welfare indicators for a wide range of mental experiences has been collated in this way, and we also provide a framework for other researchers wishing to scientifically validate welfare indicators in other species and contexts.

## 2. Selecting Welfare Indicators

Based on previously published information, potential welfare status and welfare-alerting indicators were listed, all of which had been demonstrated to be practically assessable in free-roaming wild horses [2,8,9,10].

For welfare status indicators, only animal-based indicators directly reflect the animal’s experiences and thus welfare state. However, in some circumstances there are scientifically robust links to support the use of an indirect indicator, which may be resource-based and sometimes more practical to measure or observe in free-roaming animals [2]. For example, drinking behaviour and indicators of cold discomfort may not be directly observable in wild animals, in contrast to metrics such as distance to water, ambient temperature, and weather conditions which are more easily quantifiable. Resource-based measures for evaluating compromises in Domains 1 and 2 are also commonly used in domestic horse assessments (reviewed by [11]). Furthermore, given that these resources (e.g., foraging and water availability, ambient temperature) are not under human control in the habitats of free-roaming animals, they are unlikely to change as quickly as they may in a captive environment. As such, we propose that there is likely to be a stronger link between those resource-based indicators and associated mental experiences in free-roaming animals than there might be for animals in captive settings. When the amount and/or quality of evidence of links between these types of resource-based indicators and validated welfare status indicators is high, we suggest that these resource-based indicators may carry similar weight to direct welfare status assessment. We have thus introduced the term ‘Quasi-welfare status’ indicators for these particular resource-based indicators.

The strength of evidence for the link between a welfare status or quasi-welfare status indicator and the mental experience that it infers should be reflected in a confidence score. This score will be lower if only resource-based quasi-welfare status indicators are used to infer the mental experience. In some situations, combinations of animal-based and resource-based measures may be most informative. For example, the combination of poor body condition (animal-based welfare status indicator) and insufficient food availability (resource-based quasi-welfare status indicator) allows more confident inference about the current experience of hunger than does poor body condition alone, which may only reflect past hunger.

Some status indicators may also be alerting indicators of the risk of a physical/functional impact in another Domain. For example, a very low body condition score (BCS) increases the risk of hypothermia (Domain 2) in the presence of low ambient temperatures due to effects of low body condition on thermoregulatory mechanisms [8]. Note that body condition score as a welfare status indicator may appear in both Domain 1 (Nutrition) and Domain 3 (Health), since at extremes of body condition (very underweight or very overweight), the associated pathophysiologic changes may give rise to various negative affective experiences in Domain 5 [3].

Different indicators may also have different temporal relationships to the mental experience generated. For example, body condition is indicative of an animal’s experience over a prolonged duration, whereas shivering is indicative of an immediate response to cold discomfort and provides no information of previous or ongoing thermal challenges [12].

Those indicators that were able to be scientifically validated are shown in Table 2.

## 3. Scientific Validation of Welfare Status Indicators

### 3.1. Indicators of Welfare Compromise (Negative Mental Experiences)

In this review, we first defined the precise nature of an indicator linked to physical/functional compromise in Domains 1 to 4 (Table 3). For example, for body condition, it is a lower-than-optimal body condition score that indicates undernutrition and a subsequent long-term energy deficit. Conversely, higher-than-optimal body condition scores indicate overnutrition. Each of these infer different mental experiences. The mental experience or experiences inferred to be aligned with the physical/functional impact were then noted, as detailed in Table 3.

The process for organising the scientific evidence linking indicators in Domains 1 to 4 to mental experiences in Domain 5 involves two steps that align with the Five Domains Model [12]. This process is summarised in Table 4. Each category of evidence is evaluated in each step to support the links and the more categories that are satisfied, the stronger the link.

The following sub-sections present the scientific evidence used for validating the links between the physical/functional indicators and the specific mental experiences they are proposed to reflect. While it is beyond the scope of this paper to discuss the scientific evidence in depth for every indicator, we have aimed to demonstrate the process, to summarise the most important evidence, and to direct the reader to additional resources where appropriate. As there is often more than one indicator associated with a particular mental experience, the scientific evidence linking each indicator to each inferred mental experience is presented.

#### 3.1.1. Domain 1: Nutrition

- *Indicators of thirst*

Detection of raised plasma osmolarity (i.e., dehydration) by osmoreceptors increases water-seeking and drinking behaviour, and drinking eliminates water-seeking behaviour [13], validating the link between water-seeking behaviour/drinking (observable animal-based indicator) and raised plasma osmolarity (dehydration; physical/functional state). In horses, drinking has been shown to be elicited by inducing increases in plasma osmolarity, decreases in blood volume, and increases in plasma sodium concentration [14]. Plasma osmolality has been used as a reference to assess dehydration [15]. Affective neuroscience provides evidence of the link between water-seeking behaviour/drinking and the mental experience of thirst, via neurohormonal pathways transmitting afferent inputs from osmoreceptors to higher brain centres associated with emotions [13].

However, water-seeking or thirst behaviours can be difficult to observe in free-roaming wild animals. In domesticated horses, which can be approached, a ‘drink test’ can be performed; a bucket of water is offered to the animal, and it is noted how eagerly the animal drinks and whether they drink none, some, or most of the water [16]. Given that this cannot be performed in unapproachable free-roaming wild animals, risk of dehydration may be indirectly assessed based on the resource-based indicators of how available water sources are in relation to required frequency of drinking, based on the best available data for horses. Even in domestic horses, using the resource-based indicator of water availability is regarded as a valid, reliable, and feasible indicator for on-farm assessment [17]. As the strength of evidence between water availability and risk of dehydration and thus thirst, is strong, we have regarded water availability as a quasi-welfare status indicator.

In free-roaming horses, drinking frequency depends on water availability (reviewed by [8]). Therefore, although travelling long distances to water and drinking as little as once every 24–48 h may be routine for some wild populations, these are not optimal conditions and it is likely that such horses will, at least occasionally, experience more severe thirst and/or thirst for longer periods than horses that typically drink several times daily from readily available water sources (reviewed by [8]). Factors other than location of water sources also need to be considered, as difficulty in accessing water may occur for other reasons, such as illness or injury.

Experimentally, drinking was recorded in ponies after an elevation in plasma osmolality of 3 mmol/L, which was induced after 19 h of water deprivation [14], suggesting for these animals that the mental experience of thirst became significant by 19 h of water deprivation. However, the environmental conditions during this study were not described, so it is not clear what influence ambient temperature had on the speed of onset and magnitude of dehydration. The onset of dehydration is faster when ambient temperatures are high, during exercise, lactation and when grass water content is low [8]. Accordingly, these factors need to be considered as additional welfare-alerting indicators when evaluating the impacts that the frequency of water access may have on the inferred intensity of the thirst horses may experience when their access is impeded by distance or water shortages.

- *Indicators of hunger*

Hunger is the negative mental experience that motivates food-seeking and ingestive behaviours [18]. A low body condition score (i.e., being thin or emaciated) is widely used as a welfare indicator in many species, including horses [19,20,21,22,23]. Body condition scoring has been previously discussed in depth (reviewed by [8]), as has the validation of a low BCS as an indicator of hunger [12]. Since it may take several days of food deprivation for horses and many other species to lose significant body fat, it is usually suited to indicating longer-term hunger rather than lack of satiation at a given point in time.

Step 1 in the validation is to explore the link between BCS and energy reserves/nutritional status. BCS has been shown to accurately reflect the physical bulk of fat and muscle cover, thereby providing a repeatable indication of available energy reserves related to an animal’s nutritional status, confirming the link to physical/functional impacts in Domain 1 [19,24,25]. In other mammal species, animals with a low BCS have also been shown to exhibit low blood glucose, leptin, insulin-like growth factor 1, and higher free fatty acid concentrations, indicating mobilisation of fat reserves and catabolism of muscle (reviewed by [12]).

Step 2 focusses on evaluating evidence regarding the link between energy/nutritional status and the mental experience of hunger. The strength of motivation to obtain food is one measure of this, with the expectation that animals will work harder to obtain food when they are hungrier [26]. Obtaining sufficient energy has been shown to be one of the main motivators of food intake [27]. Animals with a low BCS exhibit higher feeding motivation (e.g., walking further to access food), consume more food, and spend more time eating (reviewed by [12]).

**Figure 2:**
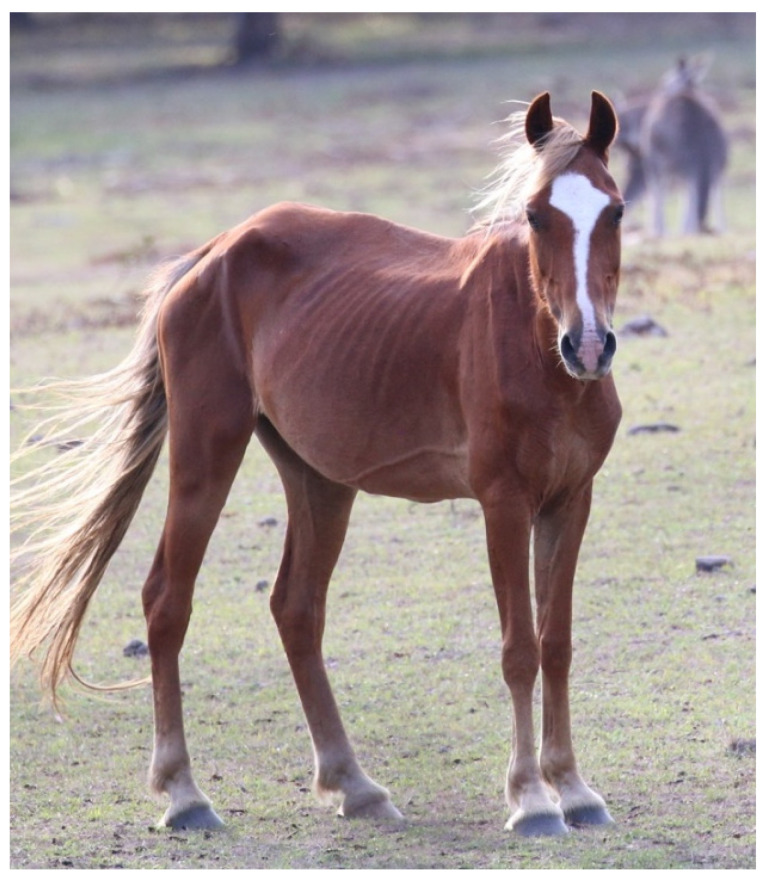
A low BCS is a scientifically validated indicator of hunger. Image: A.M. Harvey.

A low BCS typically represents undernutrition, which can be caused by insufficient quantity and quality of food (Figure 2). However, other factors can result in a low BCS even when sufficient food is available. For example, inability to access food (e.g., competition, predation risk, reduced ambulatory ability due to illness or injury), inability to ingest food normally due to oral cavity disease, interference with digestion due to intestinal disease, and increased metabolic demands resulting from disease or the heightened physiological loads of thermoregulation, pregnancy, and lactation (reviewed by [8]). Therefore, factors such as these also need to be considered as welfare-alerting indicators when assessing nutritional status. Food availability (quality and quantity) and motivation to obtain food may also provide an indication of acute hunger and could thereby enhance the use of BCS as a ‘welfare status’ indicator. However, given the challenges of accurately assessing food availability with free-roaming animals utilising large areas, taken alone, food availability is best thought of as an ‘alerting’ indicator.

#### 3.1.2. Domain 2: Physical Environment

- *Indicators of cold and heat discomfort*

In some species, including horses, detection of low or high body temperature by thermoreceptors stimulates shivering or sweating, respectively [28,29]. Acute cold exposure in climatic chambers also causes horses to shiver [30]. This validates the link between the observed indicators of shivering or sweating, and the physical/functional impact of exposure to ambient temperatures outside the animal’s thermoneutral zone. If the thermoregulatory mechanisms of shivering and sweating (or others as relevant to the species) are ineffective, then hypo- or hyperthermia can occur if cold/heat exposure persists. Humans report cold discomfort during shivering and heat discomfort during sweating, especially if associated with hypo- and hyperthermia, and resolution of these feelings of discomfort when shivering or sweating cease after ambient temperatures have returned to within the person’s thermoneutral zone. Thus, a combination of shivering and exposure to ambient temperatures below the thermoneutral zone and sweating and exposure to temperatures above the thermoneutral zone, likely reflect the mental experiences of cold or heat discomfort, respectively.

Context and environmental conditions must also be considered. Shivering due to cold may also occur in ambient temperatures within the thermoneutral zone if other factors impacting thermoregulation are present, such as a wet coat from rain or very poor body condition. In addition, tremoring with anxiety or fear can resemble shivering, whilst any process increasing adrenaline release, including pain, can result in sweating [31,32], These indicators are also relatively short lived, so they provide little or no indication of previous welfare challenges related to the physical environment, nor of ongoing welfare risks [29,30,31].

**Figure 3:**
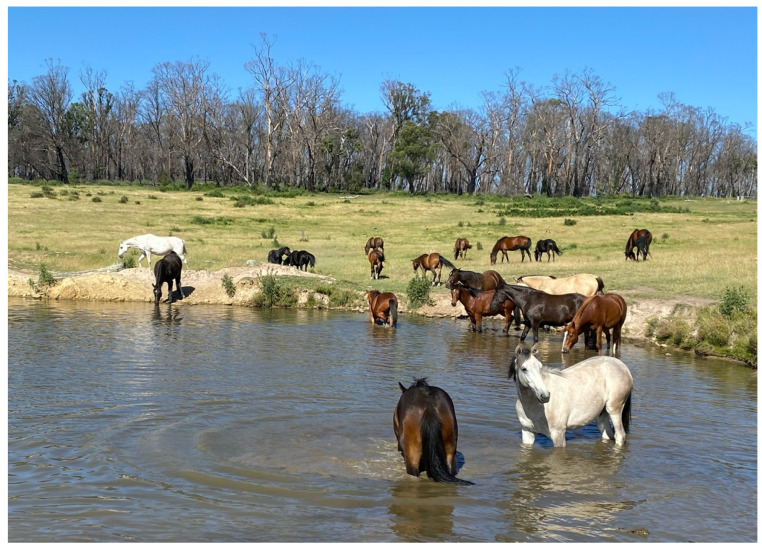
Horses exhibiting cooling behaviour by immersing themselves in cool water to mitigate heat discomfort. Image: A.M. Harvey.

Given sufficient knowledge of thermoneutral zones and thermoregulatory mechanisms, as recently reviewed [8], resource-based indicators (e.g., ambient temperature, weather conditions, presence of shelter/shade) can be cautiously used as quasi-welfare status indicators in this Domain to suggest cold or heat discomfort. With cold temperatures, seeking heat sources and shelter, and huddling may also be observed [33,34]. In windy and wet conditions horses used shelters more, particularly if the ambient temperature was less than −1 °C [34]. In warm temperatures, cooling behaviours such as moving to the shade or self-immersion in cool water may also be observed (Figure 3).

#### 3.1.3. Domain 3: Health

Indicators of health compromise have not been a focus in wild horse research to date [35]. Whilst some indicators may be extrapolated from studies of domestic horses, most health problems encountered in those horses are considered to be ‘diseases of domestication’, related to social and spatial restriction, high energy diets, and demanding work [35]. As discussed recently [8], little is known about the health status of wild horses and so optimal indicators of compromises in Domain 3 may need to be refined as knowledge advances. The mental experiences that can be best validated are different types of pain, malaise/exhaustion, and breathlessness.

*General Indicators of Pain*

Pain has been defined as ‘an unpleasant sensory and emotional experience associated with actual or potential tissue damage or described in terms of such damage’ [36]. Equine neuroanatomy and neurophysiology have long suggested that horses are able to experience physical and mental impacts of pain [37,38]. The mental experience of pain is also wide ranging in quality, intensity, frequency, and duration [39]. Complex interactions between different mental experiences may also occur. For example, ‘pain induced distress’ may give rise to a behavioural response to pain that also includes behaviours reflecting the associated distress [3,32,40].

At least 30 different types of pain have been identified [39]. For the present purposes, indicators of pain can be conveniently evaluated according to the tissues affected. Other indicators are somewhat less specific, relating to potential overlapping responses to pain, malaise, fatigue, and/or exhaustion.

*Indicators of Localised Pain*

- *Cutaneous pain: wounds*

**Figure 4:**
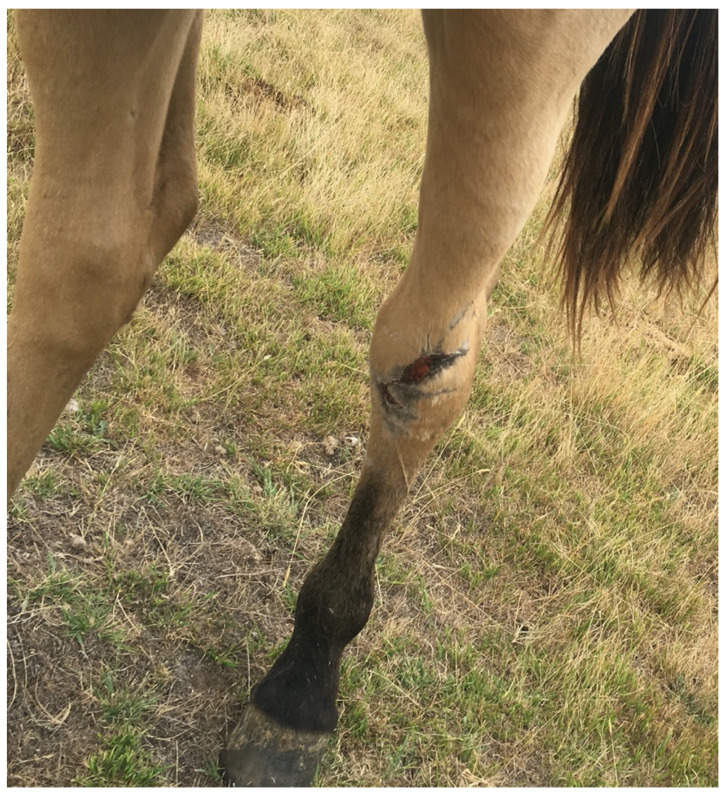
A cutaneous wound on the hindlimb of a horse. In this location, additional impacts on the underlying extensor tendon or tarsal joint may be present. The extent of such additional impacts will determine the severity of pain associated with the wound. Image: A.M. Harvey.

A cutaneous wound is any damage or break in the surface of the skin, so in this case the indicator defines the physical/functional impact (Figure 4). However, depending on the location, size, and extent of the wound, additional impacts on muscle, joints, bone, and internal organs can also be present. The presence of pain associated with wounds has been validated with nociceptive threshold testing and subsequent amelioration of behavioural responses with analgesia [41,42], thereby providing a link between the physical/functional impact and the mental experience of pain.

- *Mouth pain: Quidding and food pouching*

**Figure 5:**
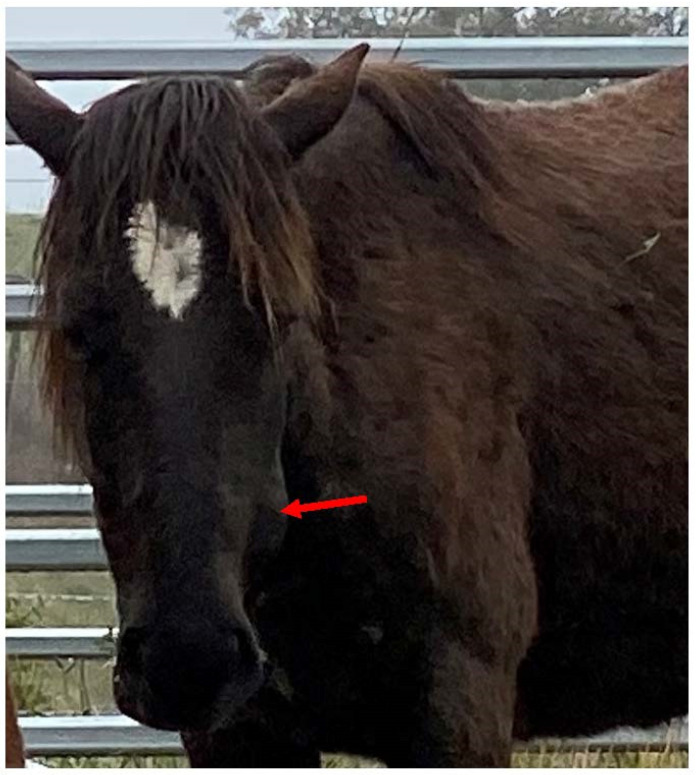
Red arrow illustrates left-sided food pouching in a captured wild young stallion. Image: A.M. Harvey.

Apart from bit-induced pain [43], mouth pain is commonly related to dental disease in domestic horses [44,45,46]. Little is known about the prevalence of dental pathology encountered in wild horses. Although it is postulated that more time spent chewing higher fibre diets with the head in a natural grazing position may lead to fewer dental disorders in wild horses compared with domestic horses, this has not yet been studied. Problematically, significant dental disease can be present in horses without externally observable clinical signs. However, the presence of quidding (dropping partially chewed food from the mouth) and food pouching in the cheeks are clear externally observable indicators of dental disorders that frequently cause secondary bruising, ulceration, and laceration of soft tissues in the mouth [44,45,46]. This information provides a link between quidding and food pouching as indicators of the physical/functional impact of oral cavity disease (Figure 5).

Physiological foundations of mouth pain in horses have recently been reviewed in detail, specifically in relation to the use of bits in domestic horses [43]. Much of the information presented on the foundations of mouth pain are also relevant to dental pain, and some descriptions of the behavioural consequences of bit pain have also been observed to be associated with dental disease [47]. The oral cavity generally has a rich supply of nociceptors susceptible to bruising, laceration, and ulceration [43,48]. Being modified periosteum, the gums are exceptionally sensitive tissues [49]. Thus, periodontal disease, dental pathology resulting in cheek ulceration, as well as dental pathology that directly exposes tooth nerve roots, would all be anticipated to cause varying types and intensities of pain. The horse grimace scale has also been used to identify pain related to dental disorders in horses [47,50,51]. Taken together, this represents compelling evidence for a link between the physical/functional impact of some oral cavity pathologies and the mental experience of pain.

- *Ocular pain: blepharospasm*

**Figure 6:**
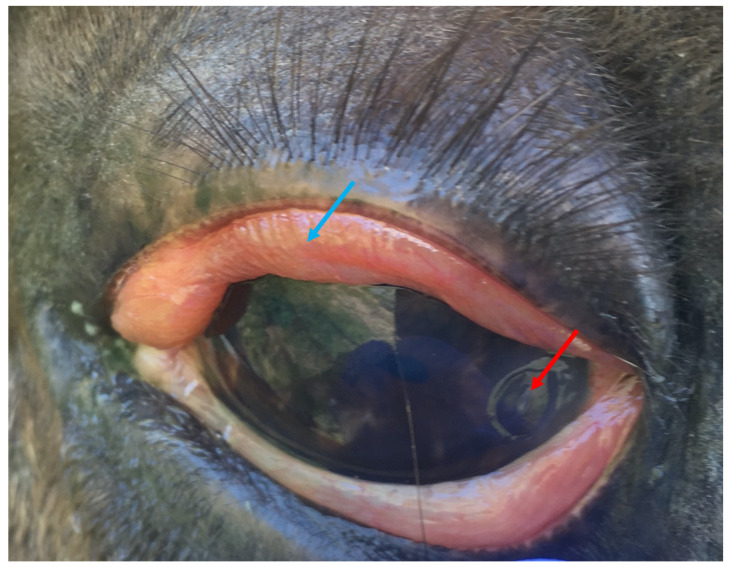
A corneal ulcer (red arrow) that was associated with corneal oedema (blue arrow) and blepharospasm, suggestive of the mental experience of acute ocular pain. Image: A.M. Harvey.

Blepharospasm is an involuntary contraction of eyelid muscles (blinking) in response to ocular pain that may arise with a range of ocular pathologies or injuries. The cornea is the most densely innervated tissue in the body, resulting in it being 300–600 times more sensitive than skin [52]. Thus, a very tiny corneal injury/ulcer can cause intense ocular pain (Figure 6). Corneal injury is the most common cause of blepharospasm in horses [53]. Stimulation of corneal nerves induces blinking, which is resolved with analgesia, thereby providing evidence of a link between corneal pathology and ocular pain.

- *Limb/foot pain: lameness*

**Figure 7:**
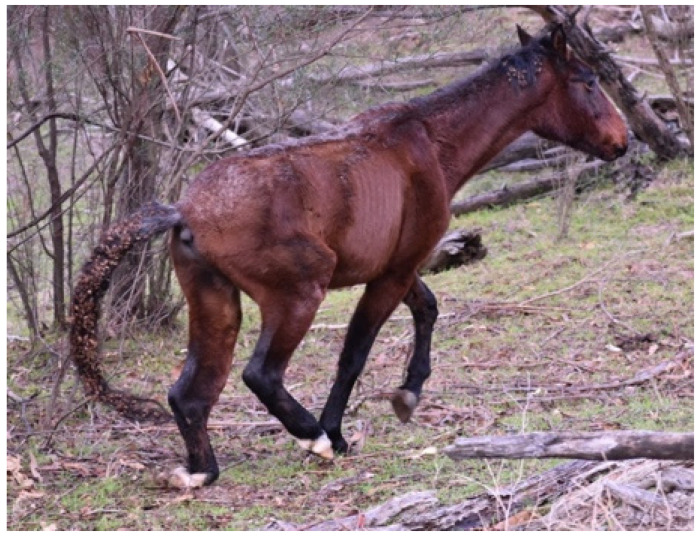
Severe injury of the lower left limb in a wild horse causing lameness, indicating presence of severe lower limb pain. Image: A.M. Harvey.

Lameness is an abnormal distribution of weight between limbs, usually associated with a change in vertical head motion in attempts to reduce pain on an affected limb. The features and severity of lameness depend on the pathology and the associated type and severity of pain [54,55,56,57,58,59,60,61,62] (Figure 7). There is extensive literature on the pathophysiology and aetiology of diseases causing lameness, with disorders of the foot being most common [21]. Investigations identifying the underlying cause provide a link between the indicator of lameness and the physical/functional impact of foot/limb pathology. Resolution of lameness with local analgesia in the form of ‘nerve blocks’ can localise the more precise anatomical location of the pathology, providing evidence of a link between the foot/limb pathology and the mental experience of pain [42,55,63].

*Indicators of Non-Specific Chronic Pain/Malaise/Fatigue/Exhaustion*

There is a wealth of research regarding the recognition of acute pain in horses [32,50,64,65], but much less regarding chronic non-specific pain and malaise [66,67]. Non-specific pain refers to the presence of pain that cannot be localised to a particular anatomical region or associated with a particular pathological process. Malaise is defined as a feeling of illness or generalised discomfort [68,69], whilst fatigue is a feeling of tiredness resulting from physical exertion or illness, with exhaustion being extreme fatigue [69].

It is recognised that aspects of behaviour that may be altered by pain include elements of demeanour, posture, and gait, as well as interactive behaviours [65]. However, behaviour is also influenced by other factors including temperament, sex, age, and environment [70]. Not showing outwards signs of pain or illness is an important survival strategy for prey species, making behavioural signs of pain or illness difficult to observe and identify in free-roaming wild horses [71]. To the authors’ knowledge, scientific assessment of pain or illness in wild free-roaming horses has not been attempted.

- *Body and ear posture*

Typical behaviours and body postures observed in both free-roaming horses and domestic horses have been well described [8,72,73]. There are challenges in detecting sickness and discomfort behaviours in horses, but body posture is important [35]. For some time, ear and tail postures and neck height have been thought to be informative about a horse’s mental state [74,75,76]. A ‘slumped’ stance has been associated with exhaustion, but this posture has not been well described [23].

More specifically, a head-lower-than-withers posture has been linked with chronic discomfort [32,35,61,71,77], as well as with more intense acute pain [64,78,79], especially when this posture is associated with the ears pointing backwards and with a ‘pain face’ grimace [32,50,50,64,71]. Intermittently moving ears backwards can occur for a range of reasons, including to aid responses to noises and agonistic social interactions [35]. However, in one large study of horses in calm environments with little external stimulation, long periods with ears held back was strongly associated with illness [35,67].

Recently, specific abnormal body postures associated with discomfort and/or exhaustion have been extensively included within a ‘horse discomfort ethogram’ to aid identification [77]. As abnormal body postures are usually recorded in association with a known cause of musculoskeletal impairment and disappear when the underlying causative process resolved [77], the link between the body posture and the physical/functional impact of musculoskeletal impairment appears valid. Furthermore, a head-lower-than-withers posture, ears back, and changes in head and tail postures have all been reported in response to variable sources of pain in domestic horses in hospital settings, with resolution of these changes in response to analgesia [32,61,64,78,79,80].

**Figure 8:**
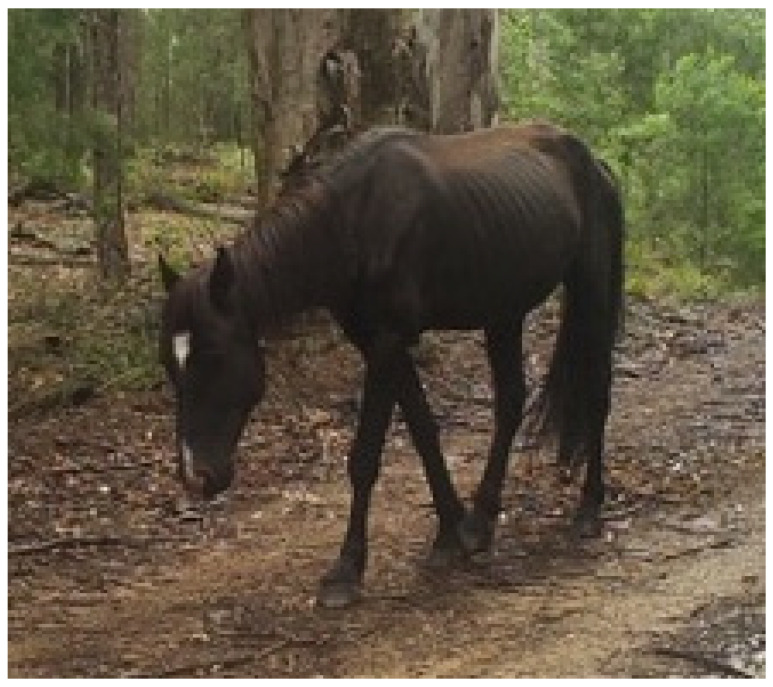
Camera trap image demonstrating head lower than withers posture in a thin wild horse, suggestive of mental experiences of chronic pain/malaise/fatigue/exhaustion. Image: A.M. Harvey.

Finally, whilst there is substantial evidence that a head-lower-than-withers posture combined with ears pointing backwards can be associated with both acute and chronic pain, this posture may also reflect the presence of exhaustion in several ways: firstly, pain-induced sleep disturbances may lead to exhaustion [81]; secondly, physical exhaustion may itself give rise to musculoskeletal discomfort [82]; and thirdly, exhaustion has also been described as a form of pain [82]. Consequently, when interpreting the head-lower-than-withers posture with ears back in affective terms, differentiating between non-specific chronic pain, malaise, fatigue, and exhaustion is currently challenging (Figure 8).

- *Facial grimace*

**Figure 9:**
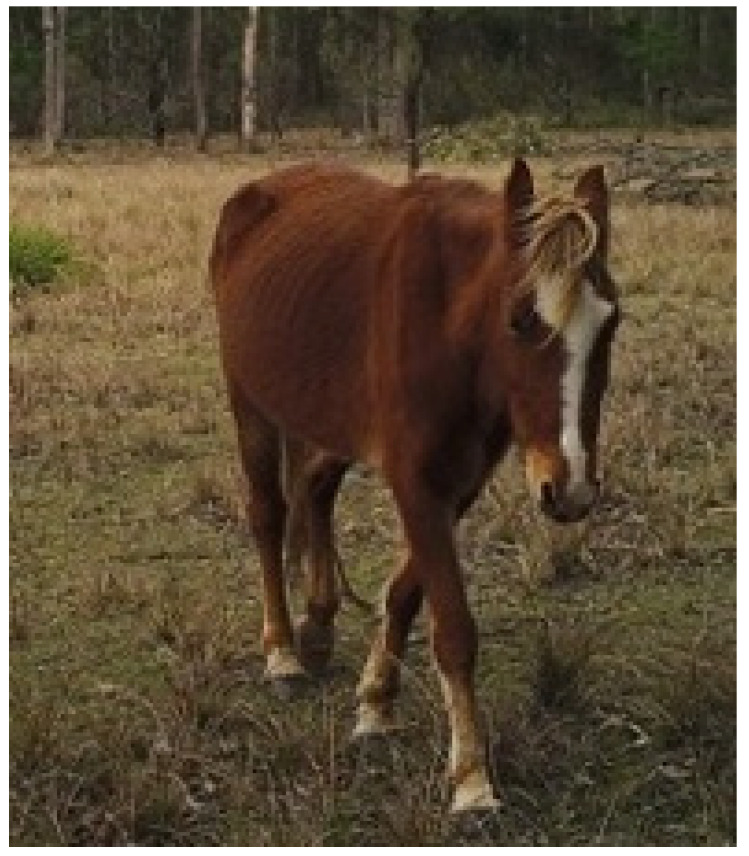
A camera trap image of a wild horse demonstrating some features of the facial grimace, suggestive of the mental experiences of pain, malaise, or exhaustion. Image: A.M. Harvey.

The equine pain grimace scale, incorporating six facial features, can provide additional insight into pain [50,51]. These features include stiffly backward ears, orbital tightening, tension above the eye area, prominent chewing muscles, strained mouths, strained nostrils, and flattening of the profile. Facial grimace features have been associated with post-operative pain [50], mouth pain [47], and musculoskeletal pain [83]. Not all of these features are observable or able to be recorded in free-roaming horses, but ear position, and, in some cases, tension above the eye and strained nostrils, can be assessed [9] (Figure 9). Note, however, that these features may not be specific to acute pain, as they may also be associated with malaise and/or exhaustion.

- *Reduced alertness/dullness/apathy*

Subjective qualitative assessment of demeanour can be very valuable [21,23,84,85,86]. Although the characteristic features of ‘reduced alertness’, ‘dullness’, and ‘apathy’ have not been clearly defined, all refer to unresponsiveness to environmental stimuli [86,87]. Body postures suggestive of apathy are also highly informative [21,23,67,85,86,87].

Apathy-associated postures have been identified in working horses in developing countries and riding-school horses, and have been correlated with presence of wounds, poor body condition, and lameness [21,85]. Lack of alertness and apathy in working equids has been strongly correlated with poor body condition, implying exhaustion, and with dermatological lesions and abnormal gaits, implying chronic pain conditions [23,85,86,87]. Reduced alertness has also been detected in combination with the ‘pain face’ grimace [50,71].

Different methods may be required to identify key features of demeanour and weakness in free-roaming horses [9]. For example, body and ear posture could be assessed from camera trap images, whereas the presence/absence of weakness and assessment of demeanour could only be performed by direct observation or assessment of camera trap videos [9].

- *Very thin/emaciated body condition*

**Figure 10:**
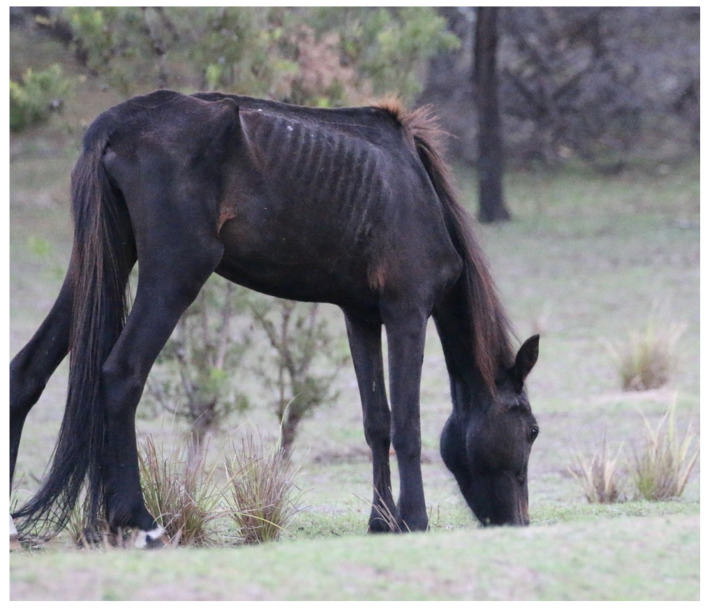
An emaciated wild horse that was observed to have both reduced activity and vigilance, suggestive of mental experiences of malaise, fatigue, and exhaustion. Image: A.M. Harvey.

Severe undernutrition, including starvation, results in a range of impairments to health including metabolic changes, muscle wasting, reduced gut mucosa integrity, immunosuppression, and increased risk of infectious diseases [8]. Reproductive efficiency and lactation are also reduced [88]. In people, the feeling of malaise is a non-specific discomfort associated with being unwell, representing a wide range of pathologies. Neuroendocrine studies have suggested an association between the feeling of malaise and cytokines altering the brain corticosteroid receptor balance [89]. This can further lead to reduced vigilance, inactivity, and feelings of depression in people. In horses, thin body condition has been correlated with lack of responsiveness and apathy [21,23,85] (Figure 10).

- *Muscular weakness*

Weakness results from a loss of muscle strength. This may be identifiable through difficulty in rising, a slow and possibly wobbly gait with difficulty lifting limbs, and difficulty lifting the head. Weakness caused by cardiovascular disease, metabolic disorders, or neurological, neuromuscular, and primary muscular disorders cannot be distinguished by direct observation alone, and not all of these will result in feelings of malaise, and/or fatigue/exhaustion. However, interpretation alongside other indicators is likely to be helpful. For example, if muscular weakness is evident in an emaciated horse, it is likely to result from low muscle mass and be associated with physical exhaustion [8].

*Indicators of Breathlessness: Increased Respiratory Rate and/or Effort*

Breathlessness is a significant animal welfare issue, with the pathophysiology previously discussed [90,91]. The term breathlessness, rather than dyspnoea and tachypnoea, has been used in animal welfare science to highlight the fact that respiration is associated not only with physical sensations, but also with affective experiences [90]. Whilst this is recognised in human medicine [92,93], the veterinary literature has typically used the term dyspnoea to describe only the physical signs of ‘difficult, laboured breathing’ [94].

The physical indicators of respiratory impairment (i.e., increased respiratory rate and effort) can arise from a wide range of pathological conditions causing different types of impairment [90]. Examples include decreased respiratory muscle function (e.g., muscle weakness due to metabolic disorders or severe undernutrition, thoracic or abdominal pain due to injuries, tissue hypoxia due to anaemia, and respiratory muscle paralysis induced by toxins such as snake bite envenomation), increased restrictive loading from conditions causing lower airway narrowing such as chronic bronchitis, disorders narrowing the upper airway, such as laryngeal hemiplegia, or reduced chest wall compliance with pulmonary diseases, such as pneumonia [90].

There may or may not be additional physical indicators of potential underlying causes for the respiratory impairment, but the resulting affective experience is still describable as breathlessness. However, in humans, different qualities of breathlessness do vary in their unpleasantness [95].

Evidence that breathlessness is an unpleasant affective experience for animals comes from the sense of urgency to engage in specific behaviours, such as withdrawal, escape attempts, struggling, and other behavioural responses to stimuli that are similar to those that cause dyspnoea in humans [13,96,97,98,99,100,101]. Electrophysiological and gene expression studies in animals also support this behavioural evidence [102,103,104]. Further, in humans, brain imaging studies demonstrate activation of cortico-limbic regions such as those activated with other unpleasant experiences including thirst, hunger, and pain [105,106,107,108], which are also known to motivate behavioural attempts to rectify the sensation [103].

#### 3.1.4. Domain 4: Behavioural Interactions

Challenges in Domain 4 result mainly from restrictions imposed by captivity and impoverished environments [3,4,5,7,81,109]. However, situations can arise in free-roaming animals that limit their ability to engage in social interactions with conspecifics, even when there are opportunities to do so. When this occurs, animals may experience feelings of loneliness and social isolation. An entire branch of neuroscience (i.e., ‘social neuroscience’) is dedicated to understanding the processes of social bonding and associated affective states [110]. In behavioural ecology, sociality has long been known to be important for fitness by its association with increasing resource acquisition, protection from predation, and by parental care increasing the survival of young [111]. The literature linking sociality to health outcomes in both people and other animals has grown rapidly over the last three decades. For example, social isolation produces such a consistent stress response that it has become an experimental model for inducing stress [112].

Negative mental experiences arise from social isolation in animals and are often referred to as ‘social pain’, in line with contemporary definitions of pain including the discomfort of any unpleasant emotional state [110]. Feelings of insecurity and fear can also be components of social pain in humans and there is behavioural evidence of this in animals [110]. Evidence suggests that animals may prioritise sociality above relieving other mental experiences in some situations. Horses expressed a high motivation and priority for any available type of social contact with conspecifics, whether it be full social contact, head contact, muzzle contact, or simply viewing another horse [113,114]. The complex social organisation of horses indicates that they are highly social animals (reviewed by [8]). Affective neuroscience provides evidence of negative affective states that occur when such social animals are kept isolated from others or are separated from familiar conspecifics [110].

**Figure 11:**
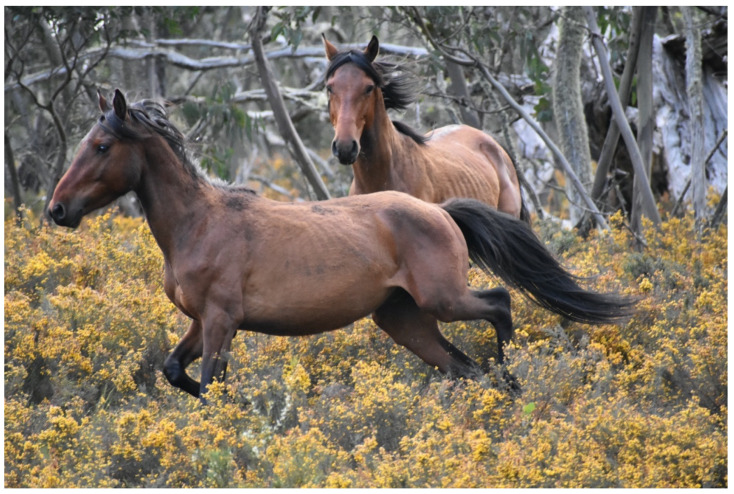
Wild horses fleeing from human disturbance, suggestive of the mental experiences of anxiety/fear/panic. Image: A.M. Harvey.

Situations in the external environment that may be perceived as threatening are also aligned with Domain 4. These include possible or actual attack by predators, separation from the protection of others, disturbance by other species (including humans), and hazardous environmental events such as flood or fire. These situations may generate negative mental experiences (Domain 5) of anxiety, fear, panic, and/or nervous vigilance [1,109,115,116] (Figure 11).

### 3.2. Indicators of Welfare Enhancement (Positive Mental Experiences)

Indicators of welfare enhancement mostly relate to observable behaviours. Positive experiences, such as pleasures associated with eating, may result from behaviours that are directed at minimising negative experiences, such as hunger. Other positive experiences may replace negative ones when there are more opportunities to engage in behaviours that the animal finds pleasurable [3,4,5,7,117,118].

Evaluating positive mental experiences requires a different approach from negative experiences, as has been described in detail elsewhere and recently summarised in relation to free-roaming animals [2,4]. For welfare enhancement in each Domain, opportunities are first evaluated and are often resource-based indicators. Utilisation of those opportunities are then assessed based on evidence of the animal using the resource. Finally, cautious judgement can be made about the type of ‘positive affective engagement’ this resource confers [119]. 

When evaluating positive mental experiences, scientific evidence for those experiences can be derived from extensive knowledge about the behaviour of the species under optimal conditions [8], and the affective neuroscience evidence of positive emotions associated with those behaviours. Under optimal conditions (i.e., where there are no or minimal welfare compromises), we can cautiously assume that free-roaming animals will experience positive affective states. This is because, unlike most domesticated forms of captivity, they have agency to engage in behaviours of their choice, such as foraging and exploration, social interactions, locomotor activity, and rest, behaviours that they are likely to find rewarding [120,121,122,123,124,125]. Thus, knowledge of welfare compromise and the study of free-roaming animals in optimal conditions provides an opportunity for learning more about those behaviours that an animal finds pleasurable.

The behaviours in each Domain that are likely to be rewarding are summarised in Table 5.

**Figure 12:**
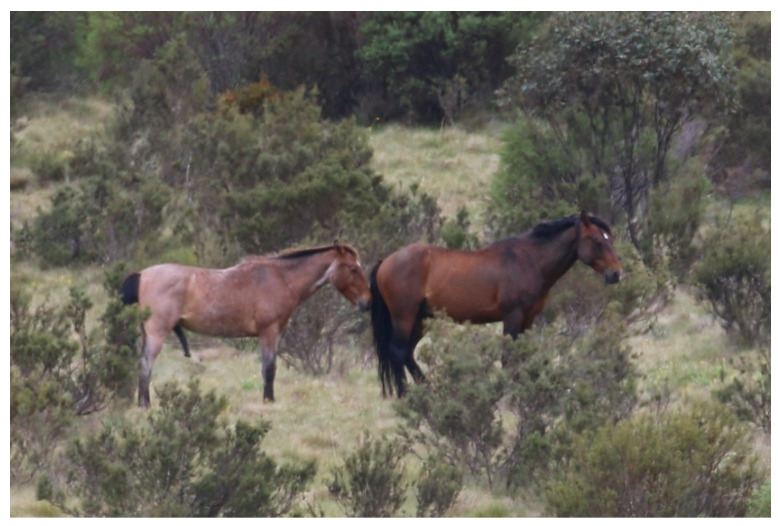
Standing resting after a period of grazing, indicative of post-prandial satiety. Image: A.M. Harvey.

Examples of positive experiences and the associated evidence from affective neuroscience are as follows:

*Domain 1:* Oral wetting and quenching pleasures of drinking, masticatory pleasures associated with eating a range of foods with varying odours, tastes and textures [120,126,127,128,129,130,131]. Resting behaviour after eating (e.g., standing-sleep) has been described as a specific indicator of post-prandial satiety in horses [132,133] (Figure 12).

*Domain 2*: Immersion in cold water and/or its evaporation from the skin, providing pleasurable cooling effects (Figure 3), and radiant heat on the skin providing pleasurable warming effects [39,134].

**Figure 13:**
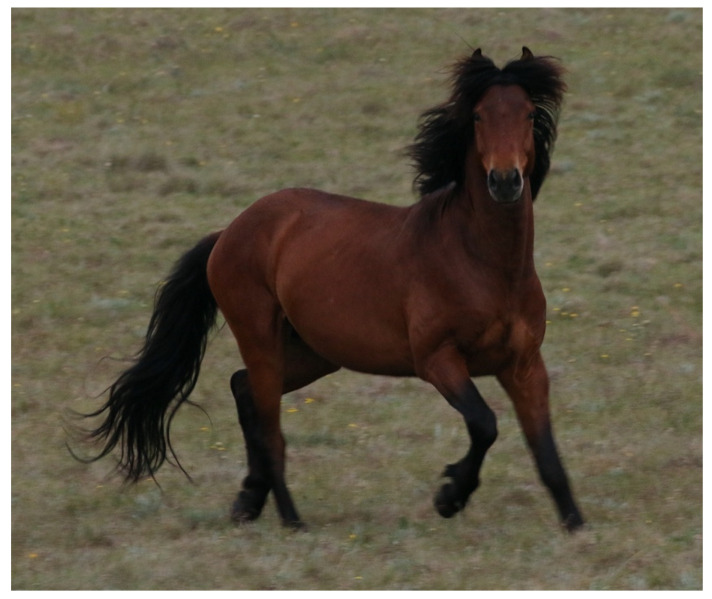
A bachelor stallion that has galloped over to investigate the photographer. The curiosity, locomotion, good health, and physical fitness may indicate vitality of fitness. Image: A.M. Harvey.

*Domain 3:* Horses are highly motivated to perform active locomotory behaviour [135,136]. When injury free, in good health, and physically fit, they can engage freely in active locomotory behaviours, which may indicate vitality of fitness (Figure 13).

**Figure 14:**
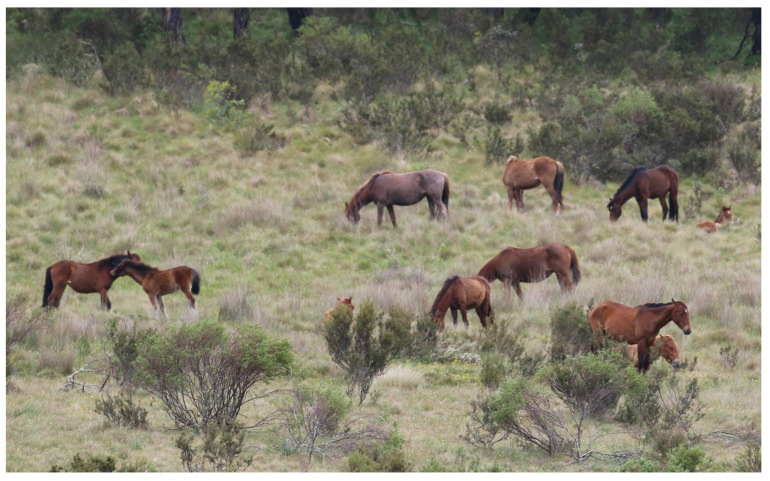
A band of wild horses demonstrating a range of rewarding behaviours, including exercise of agency, engaged foraging, standing resting, lying resting, affiliative social interactions and maternal nurturing, indicative of a range of associated positive mental experiences. Image: A.M. Harvey.

*Domain 4*: Engaging in behaviours that are rewarding [115,116,137,138]. For example, the exercise of agency including energised exploration of, and interactions with, a stimulus-rich environment [115,127,137,138], as well as focused and engaged selective foraging in environments with abundant and varied food sources [115,138,139]. Social interactions including reciprocated affiliative interactions between animals [140,141,142], the dedicated maternal nurturing and care of young [143,144,145], the joyfulness of rough-and-tumble play [125,146,147], and the eroticism and orgasmic pleasures of sexual activity [131,143,144]. The associated positive mental experiences may include, for example, feeling energised, engaged, affectionately sociable, maternally rewarded, nurtured, secure, protected, excitedly joyful, and/or sexually gratified [115,118,119,148,149] (Figure 14).

## 4. Welfare Alerting Indicators

In contrast to ‘welfare status’ and ‘quasi-welfare status’ indicators, indicators with no demonstratable direct link to a mental experience may still be useful to consider separately as ‘welfare alerting’ indicators if they provide a warning about possible current or future welfare risks [2] (Table 6). For example, low ambient temperature does not necessarily imply that all individuals are experiencing hypothermia, but it is alerting to the possibility that hypothermia may be a risk for some individuals. Likewise, there is no direct evidence that smaller band sizes and a low reproductive rate are directly associated with negative mental experiences. However, since small band sizes and low foaling rates are often associated with poor foraging availability and poor body condition, these metrics are alerting to the risk of nutritional deficiency. In some cases, combining ‘welfare alerting’ and ‘welfare status’ indicators may be helpful. For example, a high faecal egg count in the presence of diarrhoea may indicate that faecal parasites are a likely cause of the diarrhoea with affective consequences, whilst a high faecal egg count alone merely warns of a potential welfare risk [2]. As noted earlier (Section 2), some indicators of ‘welfare status’ in one Domain may also be indicators of ‘welfare risk’ in another Domain.

## 5. Discussion

Having first described indicators that are useful for assessing the welfare of free-roaming wild horses [9], this review provides the scientific evidence linking them to indicators of physical/functional states in Domains 1 to 4 and to inferred aligned mental experiences in Domain 5. This is the first time that the scientific evidence validating a comprehensive range of welfare indicators has been presented in this way. This synthesis provides an example of potential use to others who want to provide scientific support for protocols they aim to apply to other species in a range of situations.

Several reviews have evaluated a range of welfare indicators for use in domestic horses (e.g., [11,17,35,86,150]), but to date no clear distinction has been made between indicators of ‘welfare status’ (i.e., those for which scientific evidence supports their use for inferring specific mental experiences), and ‘welfare alerting’ indices. Ensuring that welfare status and welfare risk indicators are evaluated separately can improve the discriminating power of the information used to make inferences about the animals’ mental experiences. For example, a horse with cracked overgrown hooves that is not lame could be scored similarly to a lame horse with no visible hoof abnormalities in other welfare assessment protocols. However, their mental experiences would likely be different, as the first horse would not apparently be in pain, whereas the second one would be.

We have also introduced the term ‘quasi-welfare status indicator’ for a small number of resource-based indicators where there is strong evidence of a link between the resource-based indicator and a validated welfare status indicator, and thus mental experience. In these situations, the resource-based indicator may carry similar weight to direct welfare status indicators, hence the term ‘quasi-welfare status’.

To be scientifically valid, indicators need to be reliable and produce consistently repeatable results. ‘Reliability’ is established when identical physical states are documented and affective states inferred in different individuals of the same species are confronted with the same challenge or opportunity. ‘Repeatability’ is demonstrated when similar results are documented over time and by different observers [12]. These aspects of validity have not been evaluated in this paper.

Several reviews have evaluated reliability and repeatability of welfare indicators in domestic horses [17,20,21,22,23,80,85]. There are more challenges when assessing these parameters in free-roaming horses. For example, one study attempted to document reliability and repeatability of welfare indicators in free-roaming horses, but there were few welfare challenges in the horses evaluated [151]. Furthermore, these horses were habituated to people and able to be approached very closely, with only direct observations being made and assessed. This contrasts strongly with many populations of wild horses where they cannot be approached closely, precluding direct observations [9]. Any research involving assessment of welfare indicators in free-roaming wild species needs to include detailed information on the precise circumstances of the assessments, their duration and frequency, and the proximity of the assessor to the animal. The ‘feasibility’ of assessing a range of welfare indicators in free-roaming wild horses has recently been evaluated [9].

Scientifically validated indicators can be inserted into an adjusted version of the Five Domains Model, specifically designed for grading the welfare status of free-roaming wild horses, in accordance with Stages 8 and 9 of the Ten Stage Protocol. Further, ‘welfare alerting’ indices can be used for grading current and future ‘welfare risk’ as per Stage 10 of the Protocol [2]. These final stages are the subject of future publications currently in preparation, with preliminary findings already available [152].

## 6. Conclusions

This review collates, for the first time, the scientific evidence validating a range of welfare indicators for several mental experiences in horses. Whilst fewer indicators are practically able to be assessed in wild free-roaming horses compared to captive domesticated horses, we have nevertheless demonstrated that indicators can be assessed and validated for a range of negative and positive mental experiences. The negative mental experiences validated here include thirst, hunger, heat and cold discomfort, localised pain, non-specific chronic pain/malaise/fatigue/exhaustion, weakness, breathlessness, social pain, anxiety, and fear. The positive mental experiences validated here include pleasures associated with drinking, mastication, post-prandial satiety, cooling and warming, vitality of fitness, exercising agency, and participating in affiliative social interactions. This also highlights that the study of free-roaming animals under optimal conditions provides opportunity for learning more about those behaviours that an animal finds pleasurable.

We have also defined a new category of ‘Quasi-welfare status’ indicators for use in free-roaming wild animals. This new category reflects the likely stronger link between some resource-based indicators and associated mental experiences in free-roaming animals compared to captive animals, since in the former, resources are not under human control and so are unlikely to change quickly. Welfare status indicators (animal-based) are available to support inference of most of the described mental experiences in free-roaming horses. However, the combined use of welfare status/quasi-welfare status and welfare-alerting indicators provides more robust inferences. Inserting these indicators into the Five Domains Model will enable transparently valid assessment and grading of mental experiences and thus welfare status in free-roaming horses.

## Figures and Tables

**Table 1:** The Ten-Stage Protocol for assessing welfare in free-roaming wild animals (adapted from Harvey et al., 2020 [2]).

Acquire an understanding of the principles of Conservation Welfare. Acquire an understanding of how the Five Domains Model is used to assess welfare status. Acquire species-specific knowledge relevant to each Domain of the Model. Develop a comprehensive list of potential measurable/observable indicators in each physical domain, distinguishing between welfare status and welfare-alerting indicators. Select a method or methods to reliably identify individual animals. Select methods for measuring/observing the potential welfare indicators and evaluate which indicators can be practically measured/observed in the specific context of the study. Apply the process of scientific validation for those indicators that are able to be measured/observed and insert validated welfare status indicators into the Five Domains Model. Using the adjusted version of the Model that includes only the validated and practically measurable/observable welfare status indicators, apply the Five Domains grading system for grading welfare compromise and enhancement within each Domain. Assign a confidence score to reflect the degree of certainty about the data on which welfare status has been graded. Including only the practically measurable/observable welfare-alerting indicators, apply the suggested system for grading (current/future) welfare risk within each Domain.

**Table 2:** Welfare indicators that are feasible to assess in free-roaming wild horses (adapted from Harvey et al., 2021 [9]) and which have been scientifically validated in this review.

Domain	Welfare Status Indicators	Quasi-Welfare Status Indicators	Welfare-Alerting Indicators
Nutrition	Body condition score	Foraging and water availability (quality and quantity) *	Foraging and water availability (quality and quantity) Reproductive status
2. Physical Environment	Sweating Shivering	Ambient temperature and other weather conditions (e.g., humidity, sun exposure, wind, rain) in combination with shelter/shade *	Ambient temperature
			Other weather conditions (e.g., humidity, sun exposure, wind, rain, snow, hail)
			Shelter/shade
			Body condition score
3. Health	Wounds		Body condition score
	Quidding/food pouching		Hoof condition
	Blepharospasm		Coat condition
	Lameness		Skin lesions
	Body posture		Nasal discharge
	Facial grimace		Ocular discharge
	Weakness		Faecal egg count
	Qualitative assessment of behaviour (relaxed, alert, dull, apathetic)		Limb pathology
	Body condition score		
	Respiratory rate and effort		
4. Behavioural Interactions	Close spatial proximity with other horses and other affiliative interactions		Aspects of population dynamics and social organisation

* Resource-based indicators that can be cautiously used as quasi-welfare status indicators.

**Table 3:** Welfare compromise indicators, potential physical/functional negative impacts/compromises (domains one to four), and their associated inferred negative mental experience (domain five) in free-roaming horses.

Domain	Welfare Compromise (Status and Quasi-Status) Indicator	Potential Physical/Functional Impacts (Domains 1–4)	Potential Negative Mental Experience Inferred (Domain 5)
Domain 1: Nutrition	Low body condition score (±Food-seeking behaviour)	Long-term energy deficit	Long-term hunger Physical exhaustion
	* Low food availability	Short-term energy deficit	Short-term hunger
	* Water-seeking behaviour/water availability	Dehydration	Thirst
Domain 2: Physical Environment	Sweating	Hyperthermia	Heat discomfort
	* Ambient temperature above higher critical temperature ± low water availability ± humidity, ±insufficient shade		
	Shivering	Hypothermia	Cold discomfort
	* Ambient temperature below lower critical temperature, ±rainfall/wind, ±lack of shelter, ±low food availability and other factors impacting thermoregulation (e.g., very low body condition, wet coat)		
Domain 3: Health	Wounds	Disruption of integument/muscle/joints or bones	Skin/muscle/orthopaedic pain
	Quidding/food pouching	Oral cavity pathology	Mouth pain
	Blepharospasm	Ocular pathology	Ocular pain
	Lameness	Limb pathology and consequent impaired ambulatory ability	Limb pain
	Head-lower-than-withers body posture	Impaired musculoskeletal activity	Malaise, pain, exhaustion
	Facial grimace	N/A	Any pain
	Weakness	Impaired musculoskeletal activity	Malaise, exhaustion
	Reduced alertness, dull, apathetic	Systemic pathology, metabolic disturbances	Malaise, pain, exhaustion
	Very thin/emaciated body condition	Muscular weakness, metabolic disturbances	Malaise, exhaustion
	Increased respiratory rate +/or effort	Respiratory dysfunction/ impairment	Breathlessness
Domain 4: Behavioural Interactions	Isolation from other horses, no or minimal affiliative interactions	Social isolation	Loneliness, insecurity, anxiety

* Resource-based indicators where there is scientific evidence to support their cautious use as quasi-welfare status indicators, when animal-based indicators of that status are not possible or practical to assess.

**Table 4:** The two-step process for scientifically validating animal-based indicators of welfare compromise (adapted from Beausoleil and Mellor 2017 [12]).

**Step 1: Validation of the links between observed indicators and physical/functional impacts (Domains 1–4)**	Scientific understanding of the pathophysiology and aetiology of disease
	Scientific understanding of the mechanisms related to deficiency, dysfunction, disruption, or homeostatic imbalance
	Absence of elimination of the indicator using a method known to prevent or remove the underlying causative process (i.e., physical/functional impact)
	Coherence between multiple indicators in different modalities (e.g., behavioural, physiological) measured in the same situation
**Step 2: Validation of the links between physical/functional impacts (Domains 1–4) and mental experiences (Domain 5)**	Scientific understanding of the neurophysiological mechanisms underlying the mental experience in species with similar neurological capacity (i.e., affective neuroscience evidence)
	Comparison with mental experiences reported by humans in similar situations or with similar physical/functional impacts
	Elimination or reduction in a mental experience reported by humans using a method known to prevent or alleviate the physical/functional impact

**Table 5:** Examples of possible opportunities for welfare enhancement in Domains 1 to 4, indications that these opportunities are being utilised, and the inferred potential positive mental experiences (Domain 5) for free-roaming horses.

Domain	Opportunity (Resource-Based Measure)	Utilisation (Animal-Based Observation)	Potential Positive Mental Experience Inferred (Domain 5)
1. Nutrition	Sources of good-quality water readily available	Able to access and utilise water sources	Oral wetting and quenching pleasures
	Range of quality vegetation available	Observed foraging range of vegetation	Rewarding engagement of foraging, pleasures of eating a variety of food, satiety, gastrointestinal comfort
2. Physical environment	Appropriate ambient temperatures for thermal comfort, available shelter and shade Radiant sun, e.g., after a cool night. Available water source for immersion on a hot day	Utilising available shelter and shade Observed resting under radiant sun on a cool day, or immersing in water on a hot day	Thermal comfort Pleasures of radiant heat Cooling effects of immersion in cool water
3. Health	Injury free, good health and physical fitness	Observed actively engaging with environment at a range of gaits, alert to surroundings	Vitality of fitness
4. Behavioural interactions	Multiple con-specifics, both sexes, range of ages	Observed engaging in a range of affiliative social interactions on a regular basis	Affectionately sociable, maternally rewarded, contented, sense of security, social comfort

**Table 6:** Table of welfare alerting indicators for free-roaming horses in each of four physical/functional Domains detailing the current or future welfare risks that they may indicate.

Domain	Welfare Alerting Indicator	Alerts to the Risk of Future Physical/Functional Impacts (Domains 1–4)
Nutrition	Food availability	Energy/nutritional deficit
	Water-seeking behaviour/water availability	Dehydration
2. Physical Environment	Sweating	Dehydration if combined with lack of water
	Very low body condition score	Hypothermia if combined with low ambient temperatures
	Low ambient temperature, heavy rain, inadequate shelter	Hypothermia
	High ambient temperature and humidity, inadequate shade	Hyperthermia
3. Health	Very low body condition score	Illness, reduced fertility, weakness, exhaustion
	Poor coat condition	Indicators of illness
	Poor hoof condition	Impaired ambulatory ability
	High faecal egg count	Gastrointestinal pathology
	Positive *S. vulgaris* PCR	
4. Behavioural Interactions	Small band size Low reproductive rate	Social isolation

## Data Availability

Not applicable.
